# Multicenter Interventional Phase IV Study for the Assessment of the Effects on Patient's Satisfaction of Peg IFN Beta-1a (Pre-filled Pen) in Subjects With Relapsing–Remitting Multiple Sclerosis Unsatisfied With Other Injectable Subcutaneous Interferons (PLATINUM Study)

**DOI:** 10.3389/fneur.2021.637615

**Published:** 2021-04-22

**Authors:** Diego Centonze, Roberta Fantozzi, Fabio Buttari, Luigi Maria Edoardo Grimaldi, Rocco Totaro, Francesco Corea, Maria Giovanna Marrosu, Paolo Confalonieri, Salvatore Cottone, Maria Trojano, Valentina Zipoli

**Affiliations:** ^1^Scientific Institute for Research, Hospitalization and Healthcare (IRCCS) Neuromed, Pozzilli, Isernia, Italy; ^2^Synaptic Immunopathology Lab, Department of Systems Medicine, Tor Vergata University, Rome, Italy; ^3^U.O. Neurology, Multiple Sclerosis Centre, Fondazione Istituto San Raffaele “G. Giglio”, Cefalù, Italy; ^4^Demyelinating Disease Center Department of Neurology, San Salvatore Hospital - L'Aquila, L'Aquila, Italy; ^5^Neurologia, Ospedale San Giovanni Battista, Foligno, Italy; ^6^Department of Medical Sciences and Public Health, Multiple Sclerosis Centre, Binaghi Hospital University of Cagliari, Cagliari, Italy; ^7^Multiple Sclerosis Centre, Foundation IRCCS Neurological Institute Carlo Besta, Milan, Italy; ^8^Unità Operativa Complessa (UOC) Neurology, Ospedale Civico Azienda di Rilievo Nazionale ed Alta Specializzazione (ARNAS), Palermo, Italy; ^9^Department of Basic Medical Sciences, Neuroscience and Sense Organs, University of Bari “Aldo Moro” Policlinico, Bari, Italy; ^10^Biogen Italia, Milan, Italy

**Keywords:** Peg-IFN beta-1a, multiple sclerosis, adverse event, adherence, treatment satisfaction

## Abstract

Subcutaneous (SC) interferons beta (IFN-beta) are effective therapies for the treatment of relapsing–remitting multiple sclerosis (RRMS). Factors such as dosing schedule, needle intolerance/fatigue, and side effects may impact patient satisfaction with treatment. Improvement of patient satisfaction may increase the adherence to treatment and the patient quality of life. This study was aimed at evaluating the impact of switching to “Peginterferon beta-1a (Peg-IFN beta-1a)” in patients with RRMS unsatisfied with other SC interferons. The multicenter, open-label, phase IV PLATINUM study was conducted in 32 Italian centers. The primary endpoint was changes from baseline in the score of a convenience satisfaction domain of the TSQM-9 questionnaire at 12 weeks. The secondary endpoints were patients' global satisfaction, short-term adherence to treatment, satisfaction with the injection system, effect on fatigue, disease activity, and patient inability score. A total of 193 patients were enrolled and 166 (86%) completed the study, receiving Peg-IFN beta-1a for 24 weeks. Patients switching to Peg-IFN beta-1a from other SC interferons reported a significant improvement (*p* < 0.001) of Convenience Score and all other scores of the TSQM-9 questionnaire at 12 and 24 weeks (*p* < 0.001). Peg IFN beta-1a attained very high adherence to the treatment (92 and 86% at 12 and 24 weeks, respectively) with a stable annualized relapse rate (ARR). At 24 weeks, 94% of the participants were relapse free. Adverse events (AEs), recorded on 82 patients (42%), were mild or moderate. The most common AE was flu-like syndrome (29.2%). Patients switching from SC IFN beta therapy to Peg IFN beta-1a showed high treatment satisfaction with a positive safety profile, comparable with that of other currently approved first-line injectable SC interferons. This study suggests that Peg IFN beta-1a might represent a treatment choice to improve adherence in RRMS patients unsatisfied with other SC interferons.

## Introduction

Multiple sclerosis (MS) is a chronic autoimmune and neurodegenerative disorder of the central nervous system characterized by inflammation, myelin destruction, and axonal damage with subsequent oligodendrocyte and neuronal loss (1).

MS is characterized by both relapses and insidious progression, and it is notably heterogeneous in clinical course, symptomatology, and severity (2). Delayed treatment is a risk for a worse course of the disease (3, 4). Disease-modifying therapies (DMTs) are indicated to reduce relapse rates and slow disease progression for relapsing–remitting multiple sclerosis (RRMS) patients when taken as prescribed (5).

The currently available IFN-β therapies and glatiramer acetate (GA) both require either intramuscular (IM) or subcutaneous (SC) injections from as few as once a week (IFN β-1a i.m.) or once every 2 weeks (Peg IFN beta-1a) to as many as three times to four times per week (IFN β-1a SC, IFN β-1b SC, GA 40 mg) and seven times per week (GA 20 mg) (6).

Despite the benefits of injectable DMTs for MS, several problems are associated with their use, including inconvenient methods and schedules of administration, long periods of therapy, and significant side effects (7).

In particular, the high frequency of injections often produces discomfort interfering with patients' activities of daily living and may lead to refusal or inability to accept or adhere to these therapies (8). Because MS is a life-long disease, non-adherence and early treatment discontinuation can lead to greater risk for negative clinical, economics, and humanistic outcomes (9).

According to these data, satisfaction with treatment was recently incorporated into health outcomes research not only due to its value for healthcare evaluation but also for its implications in clinical practice. It is known that patients who are satisfied with their treatment show better compliance with prescriptions and play an active role in their own care (10).

Findings from the previous Phase 3 study (ADVANCE) showed that the treatment based on Peg-IFN 125 μg SC every 2 weeks is effective in RRMS, requires less-frequent injections, and is associated with a positive safety profile comparable with that of other currently approved first-line injectable therapies (11).

The PLATINUM study (NCT02587065) was aimed at evaluating the impact of switching to Peg-IFN beta-1a (125 μg SC every 2 weeks) from other injectable subcutaneous on treatment satisfaction, adherence, and other patient-reported outcomes (PROs) in an Italian real-world setting.

## Methods

Platinum was a phase IV, multicenter, single-arm, open-label study conducted in 32 Italian centers between February 3rd, 2016 and December 21st, 2017. Eligible patients were diagnosed with RRMS according to 2010 McDonald criteria, between the ages of 18 and 65 years, with an Expanded Disability Status Scale (EDSS) score between 0.0 and 5.0, and treatment with injectable subcutaneous Interferons with score <58 in the “convenience satisfaction” domain of abbreviated Treatment Satisfaction Questionnaire to Medication (TSQM; Version 1.4) (12). Exclusion criteria were pregnancy or breastfeeding, depression or other psychiatric disorders, and any contraindications to treatment with Peg-IFN-beta 1a.

Peg IFN beta-1a was supplied in a single-use, disposable, pre-filled pen. Patients started treatment by titrating with 63 mcg at the first dose, increasing to 94 mcg at the second dose, reaching the full dose of 125 mcg by the third dose, and continuing with the full dose (125 mcg) every 2 weeks thereafter. All subjects enrolled after the first period (4 weeks) of dose titration had to be treated with Peg IFN beta-1a 125 mcg (every 2 weeks) for a total period of 24 weeks.

The primary endpoint of this study was overall satisfaction with Peg IFN beta-1a treatment, as measured by the convenience satisfaction domain of TSQM at 12 weeks.

The TSQM version 1.4 is a general measure of treatment satisfaction with medication; it is made up of 14 items across 4 domains focusing on effectiveness (3 items), side effects (5 items), convenience (3 items), and global satisfaction (3 items) (13, 14).

We administered an abbreviated nine-item TSQM (TSQM-9), derived from TSQM-14 but without the five items of the side effects domain. The questionnaire utilizes a five- or seven-point Likert-type scale, and a score for each domain was calculated by summing up the corresponding items transformed on a 0–100 scale; higher values indicated higher satisfaction (15).

Secondary endpoints included changes from baseline to Week 24 on the following PRO scales: patients' adherence assessment, adapted Sclerosis Treatment Concerns Questionnaire (MSTCQ)(16), Multiple Sclerosis International Quality of Life questionnaire (MusiQoL version 5.2) (17), and Fatigue Severity Scale (FSS) (18).

Patients' adherence assessment is a questionnaire evaluating adherence over the previous 28 days and the reasons for not taking the drug at the recommended frequency of administration. Patients have been considered adherent to the prescribed dose if they answered “yes” to the first item of the questionnaire (“Have you taken the prescribed doses of your multiple sclerosis treatment correctly?”).

Adherence for Peg IFN has been evaluated using the same questionnaire (evaluation at 12 and 24 weeks). Moreover, patients who answered “no” in the second part of the questionnaire had to indicate the reasons for not taking the correct dose.

The MSTCQ is a validated 20-item patient questionnaire developed to address participant concerns with IFN-beta treatment that are not related to efficacy. It has two domains: injection-system satisfaction and side effects. The side-effects domain comprises three subscales: ISRs, global side effects, and FLS. All questions in the MSTCQ have a five-point response choice, with lower total scores indicating better outcomes. A version adapted for Plegridy has been used.

MusiQoL version 5.2 is a multidimensional self-administered questionnaire consisting of 31 items describing 9 dimensions of health-related quality of life. The nine dimensions assess many aspects of QoL that are specific to MS patients (activities of daily living, psychological well-being, symptoms, relationship with friends, relationship with family, sentimental and sexual life, coping rejection, relationship with healthcare system). Items listed in the MusiQoL questionnaire have responses describing frequency/extent of an event on a five-point scale ranging from never/not at all (option 1) to always/very much (option 5). All of the nine dimensions and the global index are linearly transformed and standardized on a 0–100 scale. Higher scores indicate a better level of health-related QoL for each dimension and for the global index score.

FSS is a specific questionnaire composed of nine statements and a visual analog scale (VAS) on the state of fatigue during the previous week. The answers are within a scale of agreement ranging from 1 to 7, with 1 representing the lowest level of agreement. An overall score of ≥36 indicates a state of fatigue.

Demographic (age, gender, education, and employment) and medical information (disease duration, previous treatments, comorbidities and concomitant therapies, the EDSS score, and the number of relapses in the previous 12 months) were obtained at baseline. All the endpoint-related questionnaires were administered at baseline and after 12 and 24 weeks of treatment.

The study was performed in compliance with the International Conference on Harmonization (ICH) and Good Clinical Practice (GCP) guidelines. It was approved by the ethics committee of the study centers, the patients had to sign a full informed consent, and all the aspects of the study were conducted according to the local laws and regulations. This trial was registered with ClinicalTrials.gov (NCT02587065) and with Clinicaltrialsregister.eu (EudraCT 2015-002201-11).

All patients who received ≥1 dose of Peg IFN beta-1a were included in the analyses. Descriptive statistics of baseline, week 12, and week 24 values of all the collected variables as well as the relevant changes from baseline were presented as mean, and standard deviations, along with median values and ranges.

A generalized linear mixed model for repeated measurements was used to evaluate the relationship between changes in a patient's total score, social-demographic factors (age, sex), and clinical characteristics (ARR, EDSS, time since MS diagnosis, treatment duration).

All analyses were performed on the Full Analysis Set (FAS) consisting of all enrolled patients who took at least one dose of the study medication. The ANOVA for repeated measures was performed to compare the changes from baseline at 12 and 24 weeks in Global Index and FSS.

## Results

A total of 193 patients were enrolled and 166 (86%) completed the study. Reasons for discontinuation were withdrawal due to adverse events (AEs) (*n* = 14), consent withdrawn (*n* = 5), non-adherence to the protocol (*n* = 3), drug interruption (*n* = 2), and others (*n* = 3).

Demographics and baseline disease characteristics of patients are summarized in Table 1.

**Table 1:** Demographic and clinical characteristics of patients switching to Peg IFN beta-1a from other injectable subcutaneous interferons.

**Characteristic**	
Patients, *n*	193
Age, mean (SD), y	42.0 (10.6)
Female, %	69.9
Race, *n* (%)	
Caucasian	192 (99.5)
Other	1 (0.5)
Education level, *n* (%)	
Primary school	2 (1.04)
Secondary school	51 (26.42)
High school	100 (51.81)
Degree	38 (19.69)
Post graduate training	2 (1.04)
Disease duration, mean (SD), y	8.4 (6.5)
Number of relapses in the past year, mean (SD)	0.2 (0.4)
EDSS, median (range)	1.5 (1–2.5)

*Data are presented as mean ± standard deviation (SD), number or count (%) where appropriate*.

Other injectable interferons taken prior to switching were SC IFN beta-1a (85.5%) and SC IFN beta-1b (14.5%) with a mean treatment duration of 5.23 ± 3.93 and 5.58 ± 3.89 years, respectively.

The reasons for not correctly taking the treatment at baseline were as follows: flu-like symptoms (59%), pain at the injection site and tiredness of injections (both 46.2%), anxiety for injection (41%), headache (37.2%), fatigue (36%), skin reactions (30.8%), weakness (26.9%), and missing dose (20.5%).

The median (IQR) EDSS remained stable at all visits without significant changes: 1.5 (1–2.5). The annual relapse rate (ARR) was 0.15 at baseline and did not show significant changes at 24 weeks (0.12).

At 24 weeks, 94% of the participants were relapse free.

Patients switching to Peg-IFN from other subcutaneous interferons reported a statistically significant improvement (*p* < 0.001) of the Convenience Score of the TSQM at 12 and 24 weeks. Mean changes of this score were 38.5 ± 23.3 (range −38.9–100) at 12 weeks and 41.9 ± 22.5 (range 0–100) at 24 weeks. Analysis for repeated measurements showed that changes in patients' convenience satisfaction are not affected by social-demographic factors (age, sex) and clinical characteristics (ARR, EDSS, time since MS diagnosis, treatment duration) (Table 2). The visits at 12 and 24 weeks were significantly associated with changes in the Convenience Score (*p* < 0.01 and *p* = 0.04, respectively, at 12 weeks vs. baseline and 24 vs. 12 weeks).

**Table 2:** Factors associated with convenience satisfaction domain.

	**coef**.	***p*-value**	**95% CI**	
T1 vs. T0	38.53	0.000	35.50	41.56
T2 vs. T0	41.93	0.000	38.85	45.01
EDSS (per 0.5 increase)	−0.08	0.797	−0.68	0.52
Age (per 1 year increase)	0.04	0.545	−0.10	0.19
Gender (female vs. male)	1.30	0.370	−1.54	4.14
Time since diagnosis (per 1 year increase)	0.03	0.822	−0.24	0.31
Duration of prior treatment (per 1 year increase)	−0.34	0.122	−0.78	0.09
Relapse in last year (per 1 increase)	−2.66	0.078	−5.61	0.30
Constant	36.65	0.000	29.18	44.13

**Figure 1:**
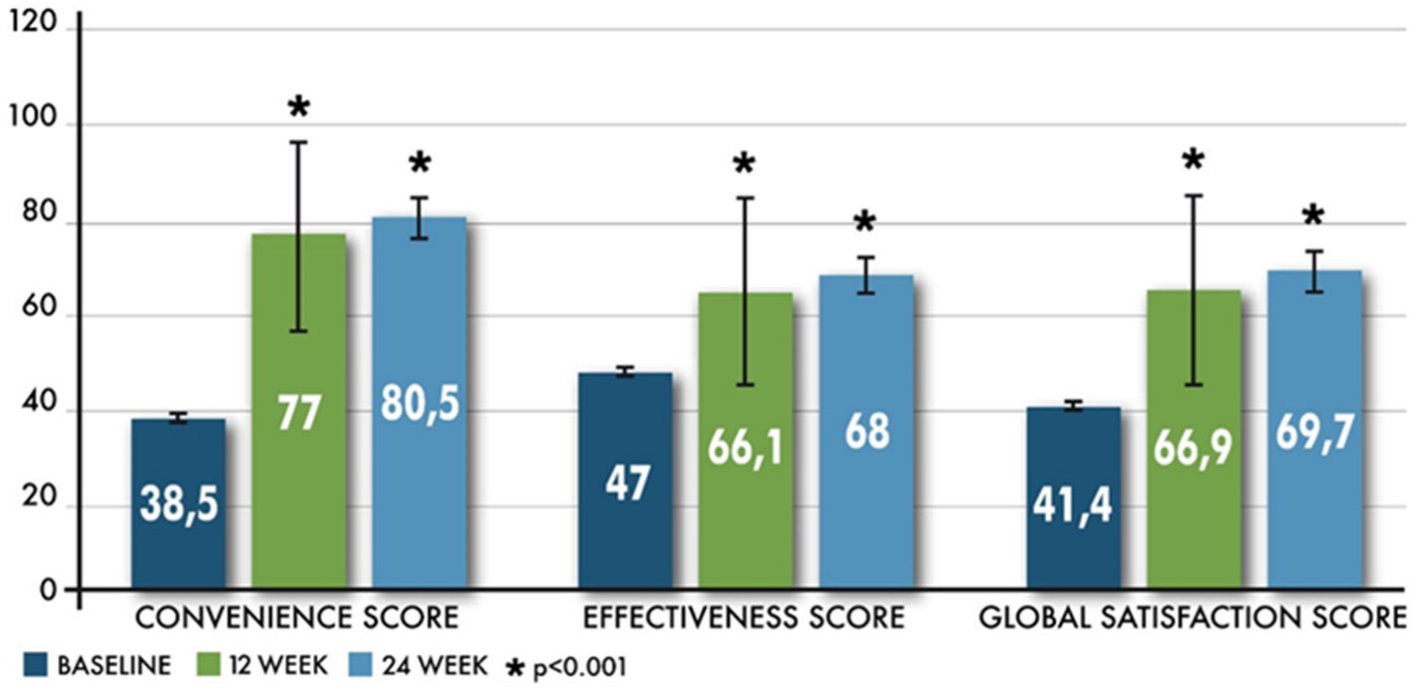
Improvement in overall TSQM scores from baseline.

A significant improvement was achieved also in the other TSQM domains (effectiveness and global satisfaction) at 12 and 24 weeks (all *p* < 0.001) (Figure 1).

A sub-analysis in three groups with a shorter duration of treatment prior to Peg IFN-beta 1a (patients with a duration of therapy prior to maximum of 2 years, 18 months, and 12 months) and considering interferons taken prior to switching to Peg IFN-beta 1a (IFN beta-1a and IFN beta 1-b) confirmed a significant increase in TSQM9 scores at all times compared to time T0.

Using the repeated measure model, even if other correction factors are included in the model (e.g., age, sex, disease duration), the duration of previous treatment did not seem to have any effect on TSQM changes.

**Figure 2:**
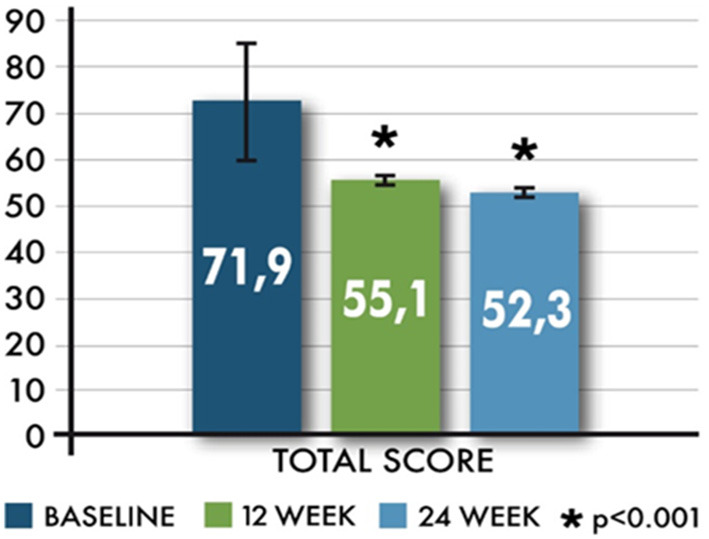
Mean (±SD) total score in MSTCQ from baseline.

The adapted MSTCQ score also significantly improved from baseline to each visit (*p* < 0.01) as shown in Figure 2.

The effect of the time (visits) on the changes of the MSTCQ total score was statistically significant; the total score decreases significantly both at 12 and 24 weeks compared to baseline (*p* = 0.012) and at 24 weeks compared to 12 weeks (*p* = 0.024); it was significantly lower for women than for men (*p* = 0.005), and it increased with increasing age (*p* = 0.048).

**Figure 3:**
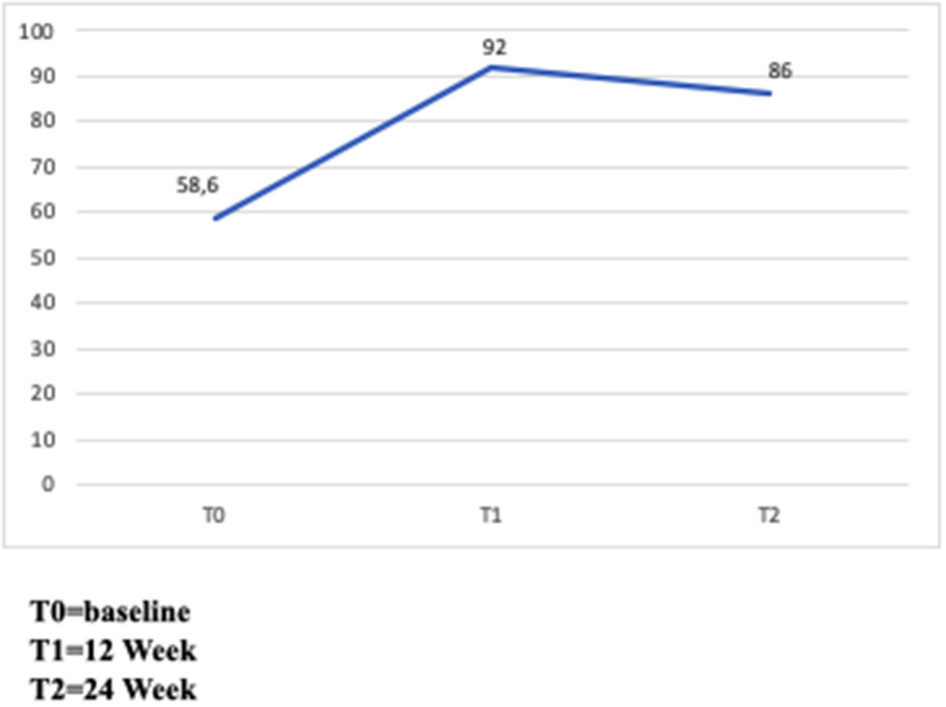
Adherence to treatment. Percentage of patients who assumed prescribed doses.

Of the 193 patients, 113 (58.6%) took the prescribed doses of other injectable subcutaneous IFNs in the previous 28 days, while 178 patients (92%) at week 12 and 166 patients (86%) at week 24 took the prescribed doses of Peg IFN (Figure 3).

The most common causes of Peg IFN missed doses were flu-like syndrome (FLS) (50%), weakness, skin reaction, headache and pain (all 20%), and fatigue (10%) at 12 weeks and fatigue (44.4%), forgetfulness and FLS (both 33.3%), weakness (22.2%), skin reaction (22.2%), and headache and pain (both 11.1%) at 24 weeks.

**Figure 4:**
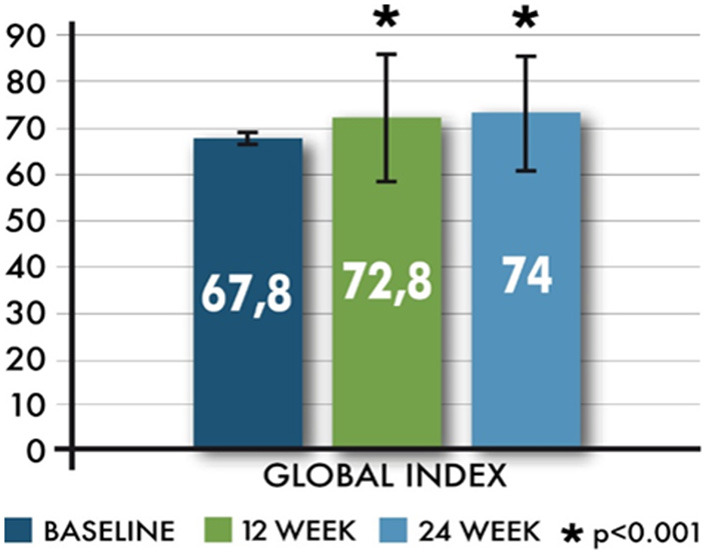
Mean (±SD) Global Index related to Multiple Sclerosis International Quality of Life.

MusiQoL total scores showed statistically significant increases from baseline to weeks 12 and 24 (*p* < 0.001). The average changes of the Global Index from baseline, at 12 and 24 weeks, were 4.6 ± 14.89 (range −40.3–56.9) and 5.0 ± 14.2 (range −34.3–57.5). The effect of the time (visits) on the changes of the Global Index was also statistically significant (*p* < 0.01) (Figure 4).

**Figure 5:**
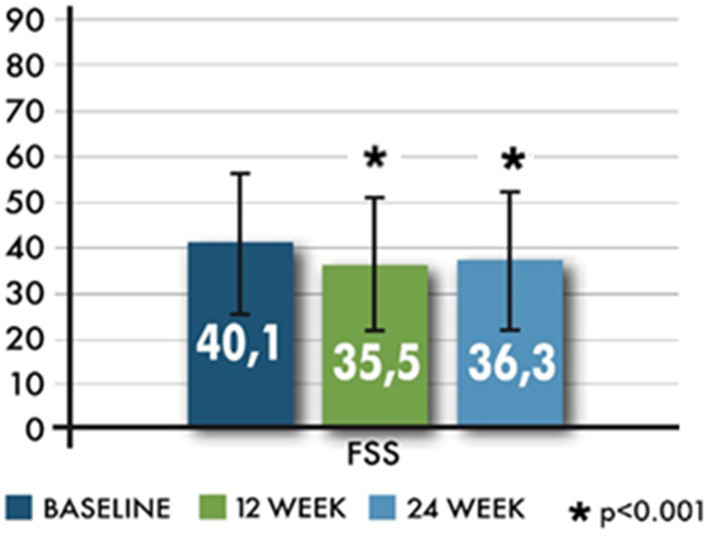
Mean changes in the Fatigue Severity Scale (FSS).

FSS total scores showed a decrease from baseline to both time points (Figure 5). FSS significantly changed at 12 and 24 weeks (T2); mean values were −4.6 ± 13.1 (range −46–33, *p* < 0.001) and −3.8 ± 13.1 (range −45–47, *p* < 0.001), respectively. The proportion of the patients scoring at least 36 in the FSS showed a significant reduction only at T1 (*p* = 0.031).

The intensity of fatigue, measured with a VAS scale (last item of FSS), was 32.2 ± 33.5 (range 0–100) at baseline, 31.4 ± 30.3 (range 0–100) at 12 weeks, and 34.2 ± 32.0 (range 0–100) at 24 weeks. No significant values were identified for comparison of VAS scores.

Overall, AEs were experienced by 42.0% of the patients who switched to Peg IFN beta-1a. AEs reported were either mild (75%) or moderate (25%). No severe AEs were reported (Table 3).

**Table 3:** Adverse events: overall treatment-emergent non-serious AEs.

	***n***	**%**
Number of subjects with a non-serious adverse event	82	42
Number mild severity adverse events	120	75
Number moderate severity adverse events	41	25
Patient with 1 adverse event	45	23.3
Patient with 2 adverse events	21	10.9
Patient with 3 adverse events	6	3.1
Patient with ≥4 adverse events	10	5.2

Forty-five (23.3%) patients reported 1 AE, 21 (10.9%) patients reported 2 AEs, 6 (3.1%) reported 3 AEs, and 10 (5.2%) patients reported 4 or more AEs. AEs leading to discontinuation were reported for 14 patients (7.3%). Study medication was responsible for 123 reported AEs (75.5%).

No serious AEs (SAEs) were experienced by the patients.

During the study, there were no pregnancies or overdoses.

## Discussion

Patient-reported outcomes (PROs) are of growing interest in research and clinical practice for understanding the effects that the disease and its treatments have on patients' lives and fast gaining influence in the choice between alternative treatments. The well-known safety profiles of IFNs allow them to be administered more confidently in patients with comorbidities and/or during pregnancy and breastfeeding. With these particular conditions, convenience may become an even more important aspect to guarantee treatment efficacy and adherence.

This open-label, phase IV study explores the convenience of assessing the effects on patient satisfaction with Peg IFN beta-1a in subjects with RRMS unsatisfied with other injectable subcutaneous interferons.

Patients switching from subcutaneous interferon beta therapy to peginterferon beta-1a every 2 weeks showed higher treatment satisfaction, higher adherence, and better quality of life with a positive safety profile, comparable with that of other currently approved first-line injectable subcutaneous interferons.

Switching to Peg IFN beta-1a improves all TSQM scores, specifically convenience scores, in patients affected by RRMS and unsatisfied with other injectable subcutaneous interferons, confirming the results from the ALLOW study (19). Considering these data, TSQM seems to be a valid patient-centered endpoint as suggested by other studies (20–22). Although FSS total scores showed a decrease between weeks 12 and 24, the intensity of fatigue seems to increase over time, and this is one of the most common causes of Peg IFN missed doses at weeks 12 and 24 (10 vs. 44.4%, respectively).

No differences were found in clinical disease activity, as assessment of confirmed EDSS and ARR variation remained stable at all visits without significant changes. Although these data are affected by a bias due to selection criteria (switch supported by dissatisfaction and not by lack of efficacy) and by a short follow-up period (24 weeks might not allow a reliable assessment of confirmed EDSS variation in a mild RR-MS population), they suggested that Peg-IFN beta-1a might have the same efficacy of other interferons.

Effectiveness scores were also increased; this suggests that patient perspectives on disease activity might be affected by treatment effects on everyday life and by the quality of life.

Adherence of about 93% is in agreement with previously reported adherence rates to subcutaneous interferon beta therapy >90% (23–25) with a higher adherence of Peg IFN beta-1a compared with other subcutaneous interferons (26). However, the relatively small sample size and the short follow-up are likely to affect the comparison with data from other IFN-beta studies with longer duration and more participants.

Finally, another limitation of this research is the lack of detailed data on previous treatments, an inherent bias of retrospective studies.

First-line injectable therapies have often a negative impact on the quality of life of patients due to uncomfortable administration methods and programs, long therapy periods and significant side effects, frequency of administration, and side effects of interferons; in this study, the greater treatment satisfaction with Peg IFN beta-1a is likely driven largely by the differences in the dosing schedule (every 2 weeks).

In conclusion, this study suggests that Peg IFN beta-1a might represent a treatment choice that is able to improve a patient's satisfaction, quality of life, and adherence, maintaining a comparable clinical efficacy in RRMS patients unsatisfied with other interferons.

## Data Availability Statement

The raw data supporting the conclusions of this article will be made available by the authors, without undue reservation.

## Ethics Statement

The studies involving human participants were reviewed and approved by IRCCS NEUROMED—Istituto Neurologico Mediterraneo. The patients/participants provided their written informed consent to participate in this study.

## Author Contributions

DC wrote the manuscript, contributed to data collection, interpretations of the results, and supervised the project. RF, FB, LG, RT, FC, MM, PC, SC, and MT contributed to data collection and interpretation of the results. VZ supervised the project. All authors contributed to the article and approved the submitted version.

## Acknowledgment

### PLATINUM Investigators

**Table:** 

**Principal investigator**	**Investigational site**	**City**
Diego Centonze	Neuromed-IRCCS Istituto Neurologico Mediterraneo	Pozzilli (IS)
Umberto Aguglia	Ospedali Riuniti A.O. Bianchi Melacrino Morelli - U.O. di Neurologia e Centro Regionale Epilessie	Reggio Calabria
Amedeo Bianchi	ASL 8 Ospedale San Donato - Centro Sclerosi Multipla - UOC Neurologia	Arezzo
Roberto Bergamaschi	IRCCS Ist. Neurologico C. Mondino - U.O.S. di Sclerosi Multipla e Malattie Demielinizzanti - Dipartimento di Clinica Neurologica e Terapie Speciali	Pavia
Vincenzo Brescia Morra	Ospedale Federico II - Centro sclerosi multipla Neurologia	Napoli
Maria Buccafusca	Policlinico G. Martino - Centro sclerosi multipla - UOC Neurologia e Malattie Neuromuscolari	Messina
Paola Cavalla	Ospedale San Giovanni Battista Le Molinette AOU Città della salute di Torino - Centro Sclerosi Multipla	Torino
Giancarlo Comi	Istituto Scientifico Universitario S. Raffaele Ospedale San Raffaele-D.I.M.E.R., Dipartimento Neurologico	Milano
Paolo Confalonieri	Fondazione Istituto Neurologico “Carlo Besta”	Milano
Francesco Corea	Nuovo ospedale S. Giovanni Battista-Struttura Complessa di Neurologia-Neuroriabilitazione	Foligno (PG)
Salvatore Cottone	A.O. Ospedali Riuniti - Villa Sofia - Cervello Centro di Neuroimmunologia	Palermo
Mario Falcini	Nuovo Ospedale di Prato, Ospedale Santo Stefano - Azienda USL 4 - Centro Sclerosi Multipla	Prato
Simonetta Galgani	Ospedale San Camillo - Forlanini Centro Sclerosi Multipla - U.O. di Neurologia	Roma
Mauro Zaffaroni	Ospedale S.Antonio Abate U.O. Neurologia 2 Centro Sclerosi Multipla,	Gallarate (VA)
Luigi Grimaldi	Fondazione Istituto San Raffaele - G. Giglio di Cefalù	Cefalù (PA)
Marco Onofri	Ospedale Clinicizzato SS Annunziata Dipartimento di Neuroscienze ed Imaging Clinica Neurologica - Università degli Studi “G. D'Annunzio	Chieti
Simona Malucchi	AOU S.Luigi Gonzaga - Centro di riferimento Sclerosi Multipla - Reparto di Neurologia	Orbassano (TO)
Giovanni Luigi Mancardi	IRCCS San Martino - DINOGMI Clinica Neurologica Universitaria	Genova
Giorgia Maniscalco	Azienda Ospedaliera di Rilievo Nazionale “A. Cardarelli” - Unità Complessa di Neurologia	Napoli
Girolama Alessandra Marfia	Fondazione PTV Policlinico Tor Vergata OU SD Centro sclerosi Multipla	Roma
Eleonora Cocco	Ospedale Binaghi Dipartimento di Neuroscienze Università di Cagliari - Centro Sclerosi Multipla	Cagliari
Massimiliano Mirabella	Policlinico A. Gemelli - Istituto di Neurologia	Roma
Ilaria Pesci	Ospedale Civile di Fidenza-Ospedale di Vaio UO Neurologia	Fidenza (PR)
Gabriella Turano	ASL CN1 - Presidio Ospedaliero di Mondovì - SC Neurologia	Mondovì (CN)
Monica Rezzonico	Ospedale Sant'Anna - UO Neurologia	Como
Giuseppe Salemi	AOU Policlinico Paolo Giaccone - Neurologia e Neurofisiopatologia	Palermo
Marco Salvetti	AO Sant'Andrea - Ambulatorio sclerosi multipla	Roma
Patrizia Sola	Nuovo Ospedale Civile S. Agostino -Estense Dipartimento Integrato di Neuroscienze - U.O. di Neurologia	Modena
Tiziana Tassinari	A.O. Ospedale S. Corona - Presidio Ospedaliero asl 2 Savonese - Divisione di Neurologia	Pietra Ligure (SV)
Rocco Totaro	Ospedale San Salvatore - Centro Sclerosi Multipla Clinica Neurologica	L'Aquila
Sebastiano Traccis	Ospedale Civile A. Segni - Az. Sanitaria Locale di Sassari - U.O. di Neurologia	Ozieri (SS)
Maria Trojano	AO Policlinico Consorziale Universitario Dip. Scienze Mediche di Base Neuroscienze ed Organi di senso	Bari

## Conflict of Interest

VZ was employed by the company Biogen Italy. The remaining authors declare that the research was conducted in the absence of any commercial or financial relationships that could be construed as a potential conflict of interest. The authors declare that this study received funding from Biogen Italia. The funder had the following involvement with the study: study design, data analysis, and revision of the manuscript.
